# Assessing the sustainability and scalability of a diabetes eHealth innovation: a mixed-methods study

**DOI:** 10.1186/s12913-023-09618-x

**Published:** 2023-06-14

**Authors:** Arani Sivakumar, Rachel Y. Pan, Angel Wang, Dorothy Choi, Ali Ben Charif, Monika Kastner, France Légaré, Catherine H. Yu

**Affiliations:** 1 Li Ka Shing Knowledge Institute, St. Michael’s Hospital, 30 Bond Street, Toronto, ON M5B 1W8 Canada; 2 VITAM - Centre de Recherche en Santé Durable, Université Laval, Quebec City, Canada; 3 Research and Innovation, North York General Hospital, Toronto, Canada; 4 Institute of Health Policy, Management and Evaluation, Dalla Lana School of Public Health, University of Toronto, Toronto, Canada; 5 Tier 1 Canada Research Chair in Shared Decision Making and Knowledge Translation, Department of Family Medicine and Emergency Medicine, Faculty of Medicine, Laval University (Québec), Québec City, G1K 7P4 Canada; 6 Department of Medicine, Faculty of Medicine, University of Toronto, Toronto, Canada; 7 Division of Endocrinology & Metabolism, Department of Medicine, St. Michael’s Hospital, Toronto, Canada; 8 Dalla Lana School of Public Health, University of Toronto, Toronto, Canada

**Keywords:** Scalability, Sustainability, Implementation, Scale-up, eHealth, Diabetes, Primary care, MyDiabetesPlan

## Abstract

**Background:**

To date, little is known about the sustainability and scalability of *MyDiabetesPlan*, an eHealth innovation designed to facilitate shared decision-making within diabetes care. To avoid the possibility of its short-lived implementation and promote wider adoption so as to promote patient-centred diabetes care, it is critical to understand *MyDiabetesPlan’*s sustainability and scalability in order to ensure its long-term impact at a greater scale. We sought to identify the sustainability and scalability potential of *MyDiabetesPlan* and its limiting factors.

**Methods:**

Using a concurrent triangulation mixed-methods approach, data were collected from 20 individuals involved in the development and implementation of *MyDiabetesPlan*. The National Health Services Sustainability Model (NHSSM) and the Innovation Scalability Self-administered Questionnaire (ISSaQ) were administered using a ‘think-aloud’ approach and subsequently, short semi-structured interviews were conducted. Mean aggregate scores and stakeholder-specific scores were generated for the NHSSM and ISSaQ, to quantitatively determine facilitating and limiting factors to sustainability and scalability. Content analysis occurred iteratively with qualitative data, to examine commonalities and differences with the quantitative findings.

**Results:**

The top facilitating factor to sustaining *MyDiabetesPlan* was “Staff involvement and training to sustain the process.”, whereas the top limiting factors were: “Adaptability of Improved Process”, “Senior Leadership Engagement” and “Infrastructure for Sustainability”. The top three facilitating factors for scale-up were “Acceptability”, “Development with Theory” and “Consistency with Policy Directives.” Conversely, the top three limiting factors were “Financial and Human Resources”, “Achievable Adoption” and “Broad Reach”. Qualitative findings corroborated the limiting/facilitating factors identified.

**Conclusions:**

Addressing staff involvement throughout the dynamic care contexts, and resource constraints impacting scale-up can enhance the sustainability and scalability of *MyDiabetesPlan***.** As such, future plans will focus on garnering organizational leadership buy-in and support, which may address the resource constraints associated with sustainability and scalability and improve the capacity for adequate staff involvement. eHealth researchers will be able to prioritize these limiting factors from the outset of their tool development to purposefully optimize its sustainability and scalability performance.

**Supplementary Information:**

The online version contains supplementary material available at 10.1186/s12913-023-09618-x.

Contributions to the literature

- This study is one of the first to concurrently evaluate both implementation science concepts (sustainability and scalability) using validated questionnaires
- This study serves as a representative case study for implementation science researchers within the primary care context
- This study also highlights the importance of simultaneously evaluating the sustainability and scalability of an eHealth tool to promote successful and ongoing implementation, rather than individually, as traditionally observed in the literature

## Introduction

‘Pilotitis’ is characterized as the proliferation of short-lived pilot eHealth projects, with a lack of consideration for its sustainability and scalability, integration into current practices and interoperability [1]. Often, this expansion of disease-specific and small-scale innovations leads to the fragmentation of such tools with limited potential for widespread community/regional use and therefore, impact at scale [2]. Previous literature has reported that up to 40% of new programs are not sustained beyond the first few years of implementation, after the termination of the initial funding [3].

Two implementation science concepts, sustainability and scalability, are often assessed independently of one another [4]. Sustainability is defined as the degree to which an innovation can be consistently used after initial implementation efforts and can be adapted to evolving contexts [4]. Scalability is known as “the ability of a health intervention shown to be efficacious on a small scale and or under controlled conditions to be expanded under real world conditions to reach a greater proportion of the eligible population, while retaining effectiveness” [5]. Innovations without sustainability and scalability capacity are limited in their ability to achieve its outcomes, and waste resources and money [6]. Formalized sustainability and scalability plans ensure that outcomes are maximized and far-reaching without compromising current processes for sustainable deployment of eHealth tools [1].

*MyDiabetesPlan* is an online patient decision-aid designed to promote shared decision-making for diabetes goal-setting in primary care by creating personalized plans for lifestyle components such as diet, exercise and medication based on personal health information (e.g., blood sugar, weight) [7]. *MyDiabetesPlan* was developed from evidence-based guidelines and was intended to complement diabetes care provision and facilitate communication between clinicians and patients [7]. From our feasibility and development studies, we found that a decision-aid such as *MyDiabetesPlan* can facilitate clinician-patient dialogue, strengthening the therapeutic relationship [8]. Furthermore, in our previous work, we conducted a 10-site randomized controlled trial (*n* = 111), which demonstrated an improvement in decisional quality and chronic illness management, with the use of *MyDiabetesPlan* [9]. Despite the demonstration of *MyDiabetesPlan*’s effectiveness, we recognize that this tool is susceptible to the challenges of sustainability and scalability beyond initial implementation.

Our previous qualitative study identified factors that were critical to optimizing the implementation conditions of *MyDiabetesPlan* through the use of the Normalization Process Theory (NPT) [10]. NPT is an implementation science theory, which seeks to examine the various stakeholder engagements at multiple levels of the healthcare system, involved in the normalization of an eHealth tool into clinical practice [11]. The use of NPT helped to identify critical factors to successful implementation of *MyDiabetesPlan* such as stakeholder buy-in, integration with clinic aspects (i.e., workflow, technology, philosophy of care), and the political climate and trends [10]. However, the extent to which *MyDiabetesPlan* had addressed or accounted for these factors was unclear. This uncertainty highlighted a gap for the present study to undertake; measuring the sustainability and scalability potential of *MyDiabetesPlan.* Specifically, it is important to assess sustainability and scalability within the context of *MyDiabetesPlan* because it can help to: (1) ensure its long-term impact and avoid pilotitis, thereby optimizing appropriate resource management and allocation; (2) support its replication in comparable settings to magnify its reach; and (3) ensure an equitable distribution of its benefits to the intended population (i.e., people living with diabetes) so as to advance diabetes population health.

The objectives of this study were to: (1) examine the level of sustainability and scalability of *MyDiabetesPlan* in its current state and (2) identify factors that limit the optimization of sustainability and scalability of *MyDiabetesPlan*.

## Methods

### Study measures

This study is part of a larger research program dedicated to integrating *MyDiabetesPlan* into primary care, in Toronto, Ontario, as described above. The purpose of this study is to evaluate the sustainability and scalability potential of *MyDiabetesPlan* as a case study, using two survey-based tools, the National Health Services Sustainability Model (NHSSM) and the Innovation Scalability Self-administered Questionnaire (ISSaQ), in order to identify the limiting factors that must be addressed to optimize implementation and scale-up within primary care. The NHSSM consists of a comprehensive and flexible framework encompassing process (i.e., monitoring progress, adaptability, credibility of benefits, benefits beyond helping patients), staff (i.e., training and involvement, behaviors, senior leaders, clinical leaders) and organizational (i.e., infrastructure and fit with goals and culture) factors [12]. The NHSSM was selected to address the sustainability of *MyDiabetesPlan* as it has been previously applied to other innovations that target an interdisciplinary care team setting and can permit a holistic understanding of a tool’s sustainability performance. The ISSaQ includes 16 scalability criteria grouped into 5 dimensions such as theory, impact, coverage, setting and cost [13]. The ISSaQ was selected as it is specifically designed for primary care; was developed within a Canadian setting; can support decision-making to prioritize this tool; and can help identify potential barriers to scalability from which strategies can be developed.

### Study design

A concurrent triangulation mixed methods approach was employed to achieve the study objectives [14]. This approach combines both qualitative and quantitative data collection and analysis to allow for us to compare and contrast findings to gain a comprehensive understanding of the phenomenon under study. In this study, participants completed the NHSSM and ISSaQ (quantitative aspect) while synchronously “thinking aloud” in the presence of the interviewer [15]. Subsequently, they answered short interview questions such as their general impressions of the questionnaire concepts in relation to *MyDiabetesPlan*, the questions’ relevance to their role, and other important aspects not considered in the questionnaires (qualitative aspects). This methodology was chosen as it not only permitted standardized input through the use of the questionnaires, but also provided participants with the opportunity to elaborate on their responses. Notably, the ‘think aloud’ qualitative component was useful for this study as it allowed participants to verbalize their reasoning and provide context when selecting questionnaire responses, and highlight potential sustainability/scalability factors they felt were important but were not incorporated into the questionnaires. This allowed for a robust and in-depth understanding of *MyDiabetesPlan*’s sustainability and scalability potential. Our study was guided by the Good Reporting of A Mixed Methods Study (GRAMMS) checklist (Supplementary Table 1) [16].

### Study population and recruitment

Participants were recruited through purposive sampling by reaching out to past and current *MyDiabetesPlan* research team members, and participants of previous studies related to *MyDiabetesPlan,* as described previously [7–10]*.* Specifically, the catchment of participants were from Ontario, as these stakeholders may be more familiar with the delivery of primary care within the context of Ontario’s healthcare system. Stakeholder categories of participants included implementation team members such as clinicians, patient partners, research team members, software development team members, Ministry of Health (MOH) representatives, and clinical/organizational leaders. This sampling approach was undertaken as it ensured that participants had a sufficient background on *MyDiabetesPlan* to answer questions regarding its sustainability and scalability on the NHSSM and ISSaQ [17].

### Data collection

Participants completed the NHSSM and ISSaQ using a “think-aloud” process with a member of the research team, which was recorded through the Zoom teleconferencing platform. In this process, participants verbally walked through the questionnaires, and provided rationales and context when selecting responses to the questionnaire items. The semi-structured interview explored the questionnaire items which they had disagreed with (i.e., aspects in which they felt that *MyDiabetesPlan* had not yet incorporated/addressed), and questionnaire items (i.e., sustainability and scalability concepts) that stood out to them within the context of *MyDiabetesPlan*.

Participant responses to the NHSSM questionnaire, which assessed stakeholder-specific factors promoting *MyDiabetesPlan*’s sustainability were measured based on a 5-item agreement scale (strongly agree, agree, neutral, disagree, strongly disagree). For each of the 10 sustainability items, a specific value was assigned for the level of agreement (i.e. strongly agree, agree, disagree, strongly disagree, not applicable), which corresponded to the values found in the original NHSSM questionnaire [18]. Responses to ISSaQ were assessed on a 6-point scale (strongly agree, agree, neutral, disagree, under evaluation and not applicable). The number of responses for each item on the scale were calculated, and a cut-off applied to indicate adequate scalability, as per the original ISSaQ questionnaire developers [13].

The primary outcomes of interest determined the level of sustainability (i.e., mean aggregate sustainability score of the NHSSM) and scalability (i.e., number of ISSaQ criteria fulfilled) attained by *MyDiabetesPlan*. Secondary outcomes of interest were the identification of the greatest limiting/facilitating factors affecting the sustainability and scalability of *MyDiabetesPlan* in its current state. These factors were identified as the constructs that received the lowest/greatest scores on both the NHSSM and ISSaQ as perceived by participants. This would be used to develop actionable items for future scale-up plans. These outcomes were assessed quantitatively and qualitatively.

### Data analysis

#### NHSSM quantitative analysis

The NHSSM model organizes sustainability into three categories (Process, Organization and Staff), with questions evaluating a total of 10 items among these categories. Questions elicited the participant’s level of agreement for whether *MyDiabetesPlan* had adequately met a particular sustainability item from the NHSSM. For each sustainability item, all participant’s agreement responses were translated to raw scores, as per the original NHSSM values. Raw scores of all participants were averaged to generate a mean aggregate score so as to order the items from the most to least satisfied. This helped to gauge the level of sustainability that *MyDiabetesPlan* had achieved for each questionnaire item. Similarly, mean scores were calculated for each stakeholder group to identify any meaningful differences among the groups. The mean aggregate score for all participants and mean scores for the individual stakeholder groups were compared to the maximum score assigned for each item, using a percent difference calculation [12]. From this, we were able to identify the sustainability items that certain stakeholders felt were optimally met (i.e. 0% difference) and the top three limiting sustainability items (i.e. largest percent differences) that should be addressed.

#### ISSaQ quantitative analysis

The ISSaQ organizes the evaluation of scalability into 5 dimensions (theory, impact, coverage, setting, cost) with a total of 16 criteria. First, we ascertained at the participant level, whether the criteria were adequately assessed, which we defined as “yes” if either strongly agree or agree were selected. Then, we ascertained at the group level whether the criteria were adequately assessed, which we defined as “yes” if over 50% of participants rated it as adequately assessed. As denoted in the literature, scalability assessment is grouped into high (i.e., innovations assessed greater than or equal to 10 criteria), medium (i.e., innovations assessed 4–9 criteria) and low (i.e., innovations assessed less than or equal to 3 criteria) [13].

#### Qualitative analysis

Inductive qualitative content analysis [19] was conducted to analyze the qualitative data. The “think-aloud” process [15] and the subsequent interview was audio-recorded, transcribed and coded by two research members (AS, RP). The codes were discussed during weekly team meetings and consensus was reached regarding discrepant codes amongst team members. The coding framework was built in an iterative fashion based on each coding session and applied to subsequent interviews. Codes were categorized into broader groups to create overarching categories. NVivo software (v.12) was used to store and organize the data.

## Results

### Participant characteristics

A total of 20 participants (7 clinicians, 2 software development team members, 3 organizational/clinical leaders, 2 ministry decision makers, 3 patients, and 3 research team members) completed the questionnaires and subsequently participated in a semi-structured interview.

### Perceived sustainability of *MyDiabetesPlan*

The mean total sustainability score amongst all stakeholders was calculated to be 69.4 against a total maximum score of 100.

**Fig. 1:**
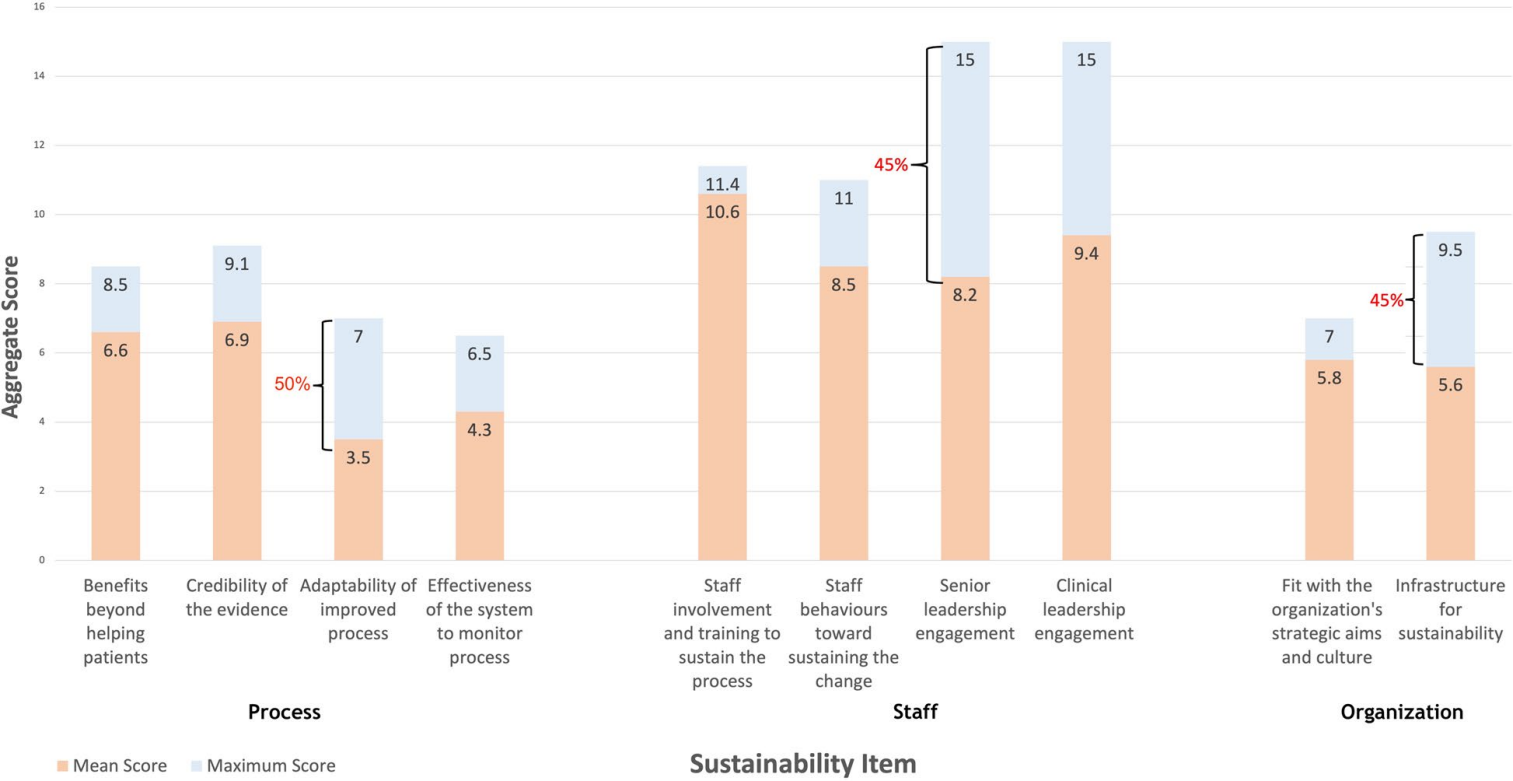
Aggregate score differences between mean stakeholder scores (*n* = 20) and maximum scores for the NHSSM for individual sustainability items

All stakeholders with the exception of organizational leaders reported that the “Staff Involvement and Training to Sustain the Process” item was the greatest facilitating factor for the sustainability of *MyDiabetesPlan* (Fig. 1). Contrastingly, responses from organizational leaders demonstrated an average percent difference of 57%, relative to the maximum score for this NHSSM sustainability item, suggesting that these stakeholders did not feel *MyDiabetesPlan* had yet optimized this item.

Aggregated mean stakeholder scores for the NHSSM identified the top three limiting factors relating to each of the main NHSSM domains: Process, Staff and Organization. These limiting factors are identified as: “Adaptability of Improved Process” (Process construct), “Senior Leadership Engagement” (Staff construct), and “Infrastructure for Sustainability”(Organization construct) (Fig. 1). These items were reported to have an average percent difference of 50%, 45%, 41% from the maximum score, respectively (Fig. 1).

Through the interviews, the majority of the stakeholder groups (13 out of 20; Non-Medical Doctors [MD] clinicians, organizational leaders, patients, research team and software development team) also identified “Senior Leadership Engagement” and “Adaptability for Improved Process” as one of the prominent limiting factors to the sustainability of *MyDiabetesPlan.* However, the reason for this rating was different, depending on stakeholder groups. Non-MD clinicians highlighted the importance of acquiring leadership support (i.e., senior leadership engagement) which they felt had not yet been fulfilled, therefore limiting the sustainability of MyDiabetesPlan. The reliance on leadership also emphasizes the relevance of adaptability to support organizational changes via clinicians (i.e., adaptability for improved process):

*“Because I’m a clinician, I need to have leadership support to implement something different in the practice [...] then we can roll it across to other clinicians, so other physicians or healthcare providers that might use the tool.”* [C005, non-MD clinician]

On the one hand, patients, research team, and software development stakeholders stated that senior leadership engagement for sustaining *MyDiabetesPlan* was relatively unimportant or not apparent to their role, which may justify their lack of agreement with these sustainability items (Table 1). In contrast, organizational leaders felt that leadership support was dependent on the leader’s area of expertise, which may justify why they felt this item was not apparent to the sustainability of *MyDiabetesPlan*:

*“If you have a leader that is non-clinical or not versed in diabetes care, it’s uncertain whether or not they’re going to push for this unless there’s a push from within, so the clinicians are pushing.”* [L002, clinical leader]

**Table 1:** Additional supporting quotes for the NHSSM limiting factors

Limiting Factor	Supporting Quotes
**Senior Leadership Engagement**	Research team participants shared: *“I’m going to say that this [senior leader support] is important but perhaps a little less important than having the staff and healthcare providers that are directly involved in using the tool”.* [C001, research team] Software development team participants also shared similar sentiments: *“This is not so relevant to me in particular because my role has a somewhat narrower scope […] I think that [senior leadership engagement] is more from the broader perspective of use within the hospital.”* [S001, software developer]
**Infrastructure for Sustainability**	*“I think in terms of infrastructure you would just need a computer, which by now most family health teams have. There are still some clinics that I think are still using paper so I guess that would impact the scalability of it.”* [C001, MD]
	*“I could see it as a product that has a long-term benefit and solution to multiple stakeholders but it still needs some tweaking […] so just kind of adapting it into our system in the healthcare system in Canada is kind of a challenge.”* [R002, research team]

In line with the quantitative findings, MD clinicians, non-MD clinicians, the MOH and research team participants also felt that “Infrastructure for Sustainability” was another one of the top three limiting sustainability factors that *MyDiabetesPlan* had not achieved. MD and non-MD clinicians assessed “Infrastructure for Sustainability” as affecting the clinical level of the healthcare system. Particularly, MD clinicians identified the reliance on paper-based systems in some clinics as a potential infrastructural barrier to the sustainability of *MyDiabetesPlan* in such settings (Table 1). However, they stated that the increased uptake in clinic technology use due to the current political climate (i.e., COVID-19 pandemic) can be leveraged to optimize future sustainability of MyDiabetesPlan:

*“We do everything on computers and then with COVID, it’s especially more amenable to virtual care. So I think that’s easy to do especially in these times, even like sharing a screen on a Zoom call.”* [C002, MD]

Furthermore, non-MD clinicians identified that the costs associated with the resources required to sustain *MyDiabetesPlan* within the clinic were not apparent, which may justify why they felt this item was not yet optimized:

*“It takes me an hour of my time and then there is still stuff to be done after that visit so it took up an entire visit. I’m not sure what the cost is for visiting or what the cost is in terms of staffing and logistics in that sense. [...] It needs to be cost-effective. Otherwise, it’s not going to work because at the end of the day money does seem to be a big factor in healthcare.”* [C005, non-MD]

In contrast, the research team and MOH participants interpreted “Infrastructure for Sustainability'' as addressing the provincial level of the healthcare system. Particularly, research team participants highlighted the challenge to adapting an eHealth innovation into the broader healthcare system (Table 1). Similarly, MOH participants also identified that the alignment of *MyDiabetesPlan* with policy directives was dependent on the current government priorities:

*“Is it consistent with what's coming out from government? Yeah, I would say so. That is important [...] but I mean that’s just dependent on what government priorities are at the time.”* [M001, MOH]

### Perceived scalability of *MyDiabetesPlan*

ISSaQ stakeholder rating results demonstrated that *MyDiabetesPlan* met 13 out of 16 criteria, indicating its high potential for scale-up.

**Fig. 2:**
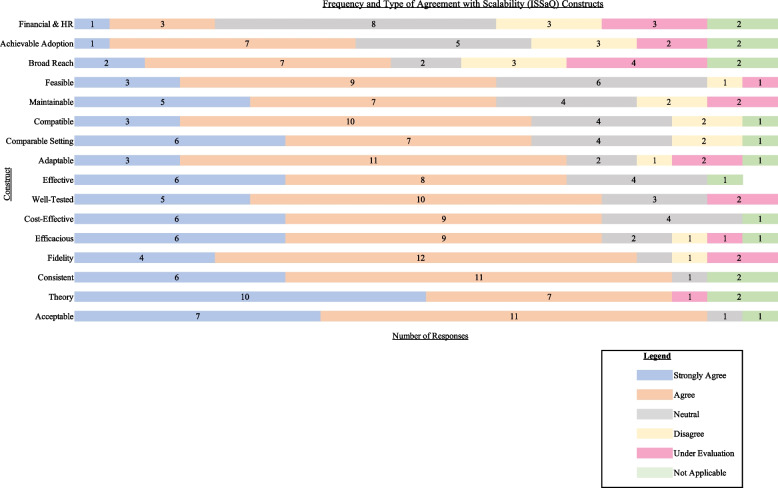
Frequency of aggregate stakeholder agreement ratings with ISSaQ statements

Figure 2 shows the results of the survey administrations ranked from lowest to highest based on overall agreement (number of strongly agree and agree). Facilitating factors (top three factors) that participants found that *MyDiabetesPlan* had achieved were: “Acceptability (Impact),” “Development with Theory (Theory)” and “Consistency with Policy Directives (Setting)”.

Quantitative findings identify two limiting factors to scalability as “Achievable Adoption (Coverage)” and “Broad Reach (Coverage).” Notably, clinicians felt that adequate coverage (i.e., achievable adoption and broad reach) were not addressed due to the issue of equity amongst different patient groups and implementing in a manner that is cognizant of such differences:

*“There may need to be some more testing from an equitable perspective within different sociodemographics or some comments on age-related measures or even income quintile access to virtual tools. [...] I think that would be important in terms of its generalizability and uptake, as well.”* [C003, clinician]

There was also confusion regarding these two constructs with stakeholders, either not understanding the individual terms, the difference between the two, or the information not being apparent to stakeholders. This was observed across the software development team, clinicians, organizational/clinical leaders, and patient stakeholders (Table 2). Research team members in particular expressed that stakeholders at a more “macro” level would be better able to speak on these coverage factors:

*“I feel like it might be better for someone else in a different role than mine, to be able to confidently and maybe in an unbiased way to answer that one; someone who deals with things at a macro level, because in my research position, it's with KT [knowledge translation] with the specific health innovation, it's a little bit more micro, right? For example, if it was someone who is like a chair of Diabetes Canada or something, or maybe someone in the Ministry who actually deals with maybe multiple interventions and can provide a different perspective on something like this, I think they would support the statement a little bit better.”* [R002, research team]

**Table 2:** Additional supporting quotes for ISSaQ limiting factors

Limiting Factors	Supporting Quotes
*Coverage*: Achievable Adoption & Broad Reach	*“MDP has demonstrated broad reach into real world settings. I do not know. I do not know—so I’m going to say it’s under evaluation—I just don’t know.”* [S001, software team] *“I think not sure what the difference is between 1 and 2 [referring to broad reach (1) and achievable adoption (2)]. Yeah I would say 4, because it reads the same to me.”* [L002, organizational/clinical leader]
*Cost*: Financial and Human Resources	*“I think some of the cost effectiveness and the human resource and financial planning around implementing a tool is a little bit, as I mentioned, outside my role as a clinician.”* [C002, Clinician] *“Yeah, so that’s not very clear to me, so I’m just going to say neutral. How important do you think this item is to you in your role. It’s important, but as I said, I put neutral because it’s not very clear.”* [P002, patient]

Our quantitative findings also report the “financial and human resources (cost construct)” as a limiting factor. This was corroborated through qualitative findings. Many stakeholders either disagreed with statements addressing the costs associated with *MyDiabetesPlan* or felt that this information was not apparent/relevant to them. For clinicians, both MD and non-MD, while they found financial and human resources related costs to be important, they did not find it to be relevant to their role (Table 2). Organizational/clinical leaders and patients similarly thought that financial- and personnel-related information were important, but this information was not apparent to them. Specifically for organizational/clinical leaders, there was an expressed desire to have more data on this subject from *MyDiabetesPlan* in the future:

*“I will stress to them they add the piece around the financial feasibility of that and they and the manpower and resources required to do that.”* [L003, Organizational/clinical leader]

However, research team members also were not aware of the financial cost of *MyDiabetesPlan* indicating that this could be an area for potential next steps for *MyDiabetesPlan:*

*“I would say it’s not clear to me in terms of the financial. I think we’ve been focusing on what is necessary for the clinicians in terms of what they need to learn, how it fits into organizational primary care setting but in terms of the financial aspect, I don’t think that’s been studied.”* [R003, research team]

## Discussion

By applying the NHSSM and ISSaQ questionnaires to *MyDiabetesPlan*, we found it to be both sustainable and scalable, and were able to identify pertinent facilitating and limiting factors to guide *MyDiabetesPlan*’s future implementation efforts. The mean aggregate score for the NHSSM was calculated to be 69.4. The majority of the participants (with the exception of organizational leaders) reported the “Staff Involvement and Training to Sustain the Process” item as the greatest facilitating factor to the sustainability of *MyDiabetesPlan*. However, the top limiting factors to sustainability were identified as: “Adaptability of Improved Process”, “Senior Leadership Engagement” and “Infrastructure for Sustainability''. The results of ISSaQ showed that *MyDiabetesPlan* met 13 out of 16 criteria, indicating its high potential for scale-up. For the ISSaQ questionnaire, the greatest facilitating factors were found to be “Acceptability (impact),” “Development with Theory (theory)” and “Consistency with Policy Directives (setting).” However, the greatest limiting factors were “Achievable Adoption (coverage)”, “Broad Reach (coverage)” and “Finances and Human Resources (cost).”

### NHSSM

Preliminary evidence suggests that a mean aggregated score equal to or above 55 indicates that the intervention “offers reason for optimism” (i.e., is above the threshold to be considered as a potentially sustainable intervention) [18]. The mean aggregated score for the NHSSM was 69.4 which is above this threshold, suggesting that *MyDiabetesPlan* is highly sustainable. However, the identification of the factors limiting the sustainability of *MyDiabetesPlan* may further explicate the mean aggregate score and how the sustainability potential can be optimized.

Respondents identified “Senior Leadership Engagement” as a limiting factor to sustainability as it was given a relatively low aggregate mean sustainability score (Fig. 1). From this, it may appear that *MyDiabetesPlan* has not sufficiently addressed this sustainability item. However, our interview data may explain this disconnect: several stakeholders felt that due to the limited scope of their role, their engagement was irrelevant or that leadership engagement and buy-in was dependent on the leader’s clinical expertise. This underlines the importance of garnering context-specific leadership support, as it can enhance the sustained implementation of evidence-based practices [20]. It also suggests the need for stakeholders to gain more role clarity by providing clear guidance on their responsibilities, so they can better understand how they can contribute to the success of *MyDiabetesPlan* and feel more inclined to participate.

Several clinician participants of our study also stated that leadership support is critical for the buy-in and dissemination of *MyDiabetesPlan* to other clinicians. Previous studies highlighted the importance of arranging training for eHealth implementation in order to improve senior leaders’ information technological competence and confidence as this influences the adoption of eHealth innovations by other clinicians and colleagues [21]. This builds the capacity for these leaders to facilitate the change management process by providing guidance to other clinicians and generate enthusiasm, and can lend to the tool’s credibility and legitimacy, which can facilitate further buy-in and adoption. Training may also address the ostensible connection between a leader’s clinical expertise (or its lack thereof) and tool buy-in as previously identified by several stakeholders. Therefore, the development of an eHealth-specific training program for senior leaders should be the next priority for *MyDiabetesPlan*.

By improving the capacity for senior leadership engagement through the identification of context-specific leaders and the establishment of training programs, *MyDiabetesPlan* may also be able to enhance the “Adaptability of Improved Process” and “Infrastructure for Sustainability” items, which were also identified as limiting factors by participants. The “Adaptability of Improved Process” item pertains to the ability for *MyDiabetesPlan* to be sustained despite internal organizational pressures (e.g., if specific individuals/groups left the project). By improving senior leadership support, more diabetes clinicians would be trained in routinely employing *MyDiabetesPlan*, thereby improving personal infrastructure and the tool’s adaptability to current organizational conditions and can facilitate succession and personnel turnover*.*

### ISSaQ

In the original ISSaQ study, 33 implementation teams within primary care were requested to complete the questionnaire based on the innovation they had developed. They found that the most assessed criteria were theory, impact, cost, setting and coverage (in order of most to least assessed) [13]. This aligns with our findings as the most assessed criteria in *MyDiabetesPlan* also included theory, impact and setting (in order from most to least). However, our findings differ in that while cost was one of the most assessed in the original ISSaQ study, it is one of the least assessed in *MyDiabetesPlan*, suggesting that the tool had not yet explicitly incorporated this item into consideration for implementation. This may be due to the nature of this project; being an eHealth innovation, it may have lower anticipated cost needs than other tangible innovations as it requires minimal resources comparatively [22].

These factors are further validated by comparing our findings to those of an eHealth innovation that has been successfully scaled across different provinces: the Champlain BASE eConsult service. They found 4 factors that were critical to the success of spread of this innovation: “(1) identifying population care needs and access problems; (2) engaging stakeholders who were willing to roll up their sleeves and take action; (3) building on current strategies and policies; and (4) measuring and communicating outcomes.” [23]. Our findings show that while *MyDiabetesPlan* achieved factors 2 and 3, factors 1 and 4 were not achieved. Thus, in comparing *MyDiabetesPlan* to another Canadian eHealth innovation that is further along in its scale-up journey, there are several areas for potential growth moving forward.

### Strengths and limitations

There was a relatively small sample size of each stakeholder group in this study, which may limit the applicability of the findings to the context of primary care. However, the occupational diversity of the stakeholders that participated in the study provided a multitude of different perspectives to inform *MyDiabetesPlan* sustainability and scalability assessments. These varying perspectives are imperative as the assessment of sustainability and scalability is inherently complex and involves the consideration of a wide range of factors. The use of validated scales to address sustainability and scalability also ensured that evidence-based constructs were acknowledged in our evaluation, which increases the robustness of the study.

The use of mixed methods permitted the triangulation of quantitative data with qualitative interview data which allowed the qualitative findings to further complement and supplement the quantitative results. This approach allowed for the exploration of perspectives that were not captured quantitatively via the two questionnaires. This study brought forth a new understanding to the *MyDiabetesPlan* research team on the tool’s current state of sustainability and scalability and future steps to optimize these two implementation concepts. This study is also one of the few to concurrently employ validated sustainability and scalability measures to evaluate an eHealth innovation. Therefore, it can be used as a representative case study for future researchers, to guide their process evaluations.

## Conclusion

Healthcare settings can be dynamic and complex, often fraught with competing priorities, placing constraints on staff and resources. Staff play a key role in the adoption of eHealth tools within the diabetes care setting, and with their buy-in, can provide valuable feedback on how the tool can be better integrated and identify opportunities for better resource allocation or optimization. The limiting factors primarily pertaining to staff involvement and resources constraints served to guide the *MyDiabetesPlan* research team in creating specific actionable items for scale-up plans, which must be addressed so as to improve implementation conditions and promote the long-term uptake of this eHealth tool. eHealth researchers can also use the concurrent approach of assessing sustainability and scalability as an evaluative framework. Notably, they can incorporate these findings within their methodology (e.g., intentional inclusion of staff perspectives and understanding the impact of resource constraints), study design (e.g., incorporating sustainability and scalability concepts), intervention development (e.g., ensuring there is opportunity for adaptations to dynamic care contexts), and knowledge translation of findings.

## Supplementary Information

**Additional file 1:  Supplementary Table 1.** Good Reporting of a Mixed Methods Study (GRAMMS) Checklist.

## Data Availability

All data generated or analyzed during this study are included in this published article.

## Abbreviations

NHSSM: NHS Sustainability Model
ISSaQ: Innovation Scalability Self-administered Questionnaire
MOH: Ministry of Health
NPT: Normalization Process Theory
