# Supplementary material for: Assessing the sustainability and scalability of a diabetes eHealth innovation: a mixed-methods study
Source: BMC Health Serv Res. 2023 Jun 14;23:630. doi: 10.1186/s12913-023-09618-x (PMC10264878; doi:10.1186/s12913-023-09618-x)
Supplement: Supplementary file 1 — Additional file 1: Supplementary Table 1. Good Reporting of a Mixed Methods Study (GRAMMS) Checklist. [file 12913_2023_9618_MOESM1_ESM.docx]

**Supplementary Table 1:** Good Reporting of a Mixed Methods Study (GRAMMS) Checklist

| Guideline | Section and page |
| --- | --- |
| Describe the justification for using a mixed methods approach to the research question | Study design and overview (page 7) |
| Describe the design in terms of the purpose, priority and sequence of methods | Data collection (Page 8-9) |
| Describe each method in terms of sampling, data collection and analysis | Data collection (page 8-9)  Data analysis (page 9 - 11) |
| Describe where integration has occurred, how it has occurred and who has participated in it | Study population and recruitment (page 7-8)  Data collection (page 8-9)  Participant characteristics (page 11) |
| Describe any limitation of one method associated with the present of the other method | Discussion: Strengths and Limitations (page 19-20) |
| Describe any insights gained from mixing or integrating methods | Discussion: Strengths and Limitations (page 19-20) |

Guideline from: O'Cathain A, Murphy E, Nicholl J. The quality of mixed methods studies in health services research. J Health Serv Res Policy. 2008;13: 92-98.
